# Dacarbazine and interferon **α** with or without interleukin 2 in metastatic melanoma: a randomized phase III multicentre trial of the Dermatologic Cooperative Oncology Group (DeCOG)

**DOI:** 10.1054/bjoc.2001.1731

**Published:** 2001-04

**Authors:** A Hauschild, C Garbe, W Stolz, U Ellwanger, S Seiter, R Dummer, S Ugurel, G Sebastian, D Nashan, R Linse, W Achtelik, P Mohr, R Kaufmann, M Fey, J Ulrich, W Tilgen

**Affiliations:** Departments of Dermatology, Christian-Albrechts-University; Eberhard-Karls-University, Tübingen; Franz-Josef-Strauß-University, Regensburg; University Hospital, Heidelberg; Universitäts-Spital, Zürich/Switzerland; Universität des Saarlandes, Homburg; Technical University Dresden; University Hospital Münster; Klinikum Erfurt; Medizinische Universität Lübeck; Kreiskrankenhaus Buxtehude; Johann-Wolfgang-Goethe-University, Frankfurt; Institut für Medizinische Onkologie, University Bern/Switzerland; Otto-von Guericke-University, Magdeburg

**Keywords:** melanoma, metastasis, interferons, interleukin 2, biochemotherapy

## Abstract

In several phase II-trials encouraging tumour responses rates in advanced metastatic melanoma (stage IV; AJCC-classification) have been reported for the application of biochemotherapy containing interleukin 2. This study was designed to compare the efficacy of therapy with dacarbazine (DTIC) and interferon α (IFN-α) only to that of therapy with DTIC and IFN-α with the addition of interleukin 2 (IL-2) in terms of the overall survival time and rate of objective remissions and to provide an elaborated toxicity profile for both types of therapy. 290 patients were randomized to receive either DTIC (850 mg/m^2^every 28 days) plus IFN-α2a/b (3 MIU/m^2^, twice on day 1, once daily from days 2 to 5; 5 MIU/m^2^3 times a week from week 2 to 4) with or without IL-2 (4.5 MIU/m^2^for 3 hours i.v. on day 3; 9.0 MIU/m^2^i.v. day 3/4; 4.5 MIU/m^2^s.c. days 4 to 7). The treatment plan required at least 2 treatment cycles (8 weeks of therapy) for every patient. Of 290 randomized patients 281 were eligible for an intention-to-treat analysis. There was no difference in terms of survival time from treatment onset between the two arms (median 11.0 months each). In 273 patients treated according to protocol tumour response was assessable. The response rates did not differ between both arms (*P* = 0.87) with 18.0% objective responses (9.7% PR; 8.3% CR) for DTIC plus IFN-α as compared to 16.1% (8.8% PR; 7.3% CR) for DTIC, IFN-α and IL-2. Treatment cessation due to adverse reactions was significantly more common in patients receiving IL-2 (13.9%) than in patients receiving DTIC/IFN-α only (5.6%). In conclusion, there was neither a difference in survival time nor in tumour response rates when IL-2, applied according to the combined intravenous and subcutaneous schedule used for this study, was added to DTIC and IFN-α. However, toxicity was increased in melanoma patients treated with IL-2. Further phase III trials with continuous infusion and higher dosages must be performed before any final conclusions can be drawn on the potential usefulness of IL-2 in biochemotherapy of advanced melanoma. © 2001 Cancer Research Campaign http://www.bjcancer.com


*British Journal of Cancer* (2001) **84**(8), 1036–1042
© 2001 Cancer Research Campaign
doi: 10.1054/ bjoc.2001.1731, available online at http://www.idealibrary.com on IDEAL®

### Dacarbazine and interferon α with or without interleukin 2 in metastatic melanoma: a randomized phase III multicentre trial of the Dermatologic Cooperative Oncology Group (DeCOG)

**A Hauschild[1], C Garbe[2], W Stolz[3], U Ellwanger[2], S Seiter[4,6], R Dummer[5], S Ugurel[6], G Sebastian[7], D Nashan[8], R Linse[9], W Achtelik[10], P Mohr[11], R Kaufmann[12], M Fey[13], J Ulrich[14] and W Tilgen[4,6]**

From the Departments of Dermatology: [1]Christian-Albrechts-University, Kiel; [2]Eberhard-Karls-University, Tübingen; [3]Franz-Josef-Strauß-University, Regensburg; [4]University Hospital, Heidelberg; [5]Universitäts-Spital, Zürich/Switzerland; [6]Universität des Saarlandes, Homburg; [7]Technical University Dresden; [8]University Hospital Münster; [9]Klinikum Erfurt; [10]Medizinische Universität Lübeck; [11]Kreiskrankenhaus Buxtehude; [12]Johann-Wolfgang-Goethe-University, Frankfurt; [13]Institut für Medizinische Onkologie, University Bern/Switzerland; [14]Otto-von Guericke-University, Magdeburg

**Summary** In several phase II-trials encouraging tumour responses rates in advanced metastatic melanoma (stage IV; AJCC-classification) have been reported for the application of biochemotherapy containing interleukin 2. This study was designed to compare the efficacy of therapy with dacarbazine (DTIC) and interferon α (IFN-α) only to that of therapy with DTIC and IFN-α with the addition of interleukin 2 (IL-2) in terms of the overall survival time and rate of objective remissions and to provide an elaborated toxicity profile for both types of therapy. 290 patients were randomized to receive either DTIC (850 mg/m$^2$ every 28 days) plus IFN-α2a/b (3 MIU/m$^2$, twice on day 1, once daily from days 2 to 5; 5 MIU/m$^2$ 3 times a week from week 2 to 4) with or without IL-2 (4.5 MIU/m$^2$ for 3 hours i.v. on day 3; 9.0 MIU/m$^2$ i.v. day 3/4; 4.5 MIU/m$^2$ s.c. days 4 to 7). The treatment plan required at least 2 treatment cycles (8 weeks of therapy) for every patient. Of 290 randomized patients 281 were eligible for an intention-to-treat analysis. There was no difference in terms of survival time from treatment onset between the two arms (median 11.0 months each). In 273 patients treated according to protocol tumour response was assessable. The response rates did not differ between both arms ($P = 0.87$) with 18.0% objective responses (9.7% PR; 8.3% CR) for DTIC plus IFN-α as compared to 16.1% (8.8% PR; 7.3% CR) for DTIC, IFN-α and IL-2. Treatment cessation due to adverse reactions was significantly more common in patients receiving IL-2 (13.9%) than in patients receiving DTIC/IFN-α only (5.6%). In conclusion, there was neither a difference in survival time nor in tumour response rates when IL-2, applied according to the combined intravenous and subcutaneous schedule used for this study, was added to DTIC and IFN-α. However, toxicity was increased in melanoma patients treated with IL-2. Further phase III trials with continuous infusion and higher dosages must be performed before any final conclusions can be drawn on the potential usefulness of IL-2 in biochemotherapy of advanced melanoma. © 2001 Cancer Research Campaign http://www.bjcancer.com

**Keywords**: melanoma; metastasis; interferons; interleukin 2; biochemotherapy

Despite some efforts in the adjuvant treatment of high-risk melanoma (stageII/III) (Kirkwood et al, 1996; Grob et al, 1998; Pehamberger et al, 1998), little has changed in the management of advanced metastatic disease, stage IV as defined by the American Joint Committee on Cancer (AJCC) (Buzaid et al, 1997). The median survival times of affected patients reported in various studies differ, but the most recent randomized trials arrive at survival times between 6 and 9 months (Chapman et al, 1999). In all studies up to the present survival times of more that 3 years are very rare.

If a ‘golden standard’ for the treatment of advanced melanoma existed, it would be dacarbazine (DTIC), but unfortunately DTIC is only able to produce tumour responses in 12% of treated patients and the impact on survival time remains unclear (Hill et al, 1984). Temozolomide, a novel oral alkylating agent with a broad spectrum of antitumor activity and relatively little toxicity, was recently shown to be equally effective as DTIC in the treatment of advanced metastatic melanoma (Middleton et al, 2000).

Complex combinations of cytotoxic drugs with biological response modifiers, such as interferon α (IFNα) and interleukin 2 (IL-2), demonstrated encouraging results in achieving long-lasting tumour responses (Legha et al, 1998; Richards et al, 1999). A relatively large number (about 10%) of patients with lasting complete remissions will survive for many years. However, most results were drawn from single-institutional phase II-trials with patients who were probably selected (Legha et al, 1998; Richards et al, 1999). Recently, a prospective-randomized trial compared treatment with cisplatin, DTIC and tamoxifen only to a combination of these substances with IL-2 and IFNα (Rosenberg et al, 1999). Although toxicity was increased, the addition of immunotherapy to chemotherapy did not lead to an improvement in either the response rate or median survival time. The authors concluded that chemoimmunotherapy cannot be recommended until well-designed, prospective-randomized protocols have been developed (Rosenberg et al, 1999).

*Received 18 August 2000*
*Revised 8 January 2001*
*Accepted 22 January 2001*

*Correspondence to:* A Hauschild

The rationale of the current study was to compare the efficacy and tolerability of a DTIC and IFNα combination alone to the same schedule with the addition of IL-2. At the time the study design was being developed (1993/1994) a meta-analysis (data not published) revealed a 29% remission rate for patients treated in phase II- and phase III-trials using DTIC and IFNα combined (Falkson et al, 1991; Punt, 1998). These results lead to the decision to use this combination as a kind of standard of care for advanced melanoma, despite controversial discussions and results published within this time period (Falkson et al, 1998).

To our knowledge this study represents the first randomized multi-centre trial on the effect of IL-2 as a supplement to conventional therapy. Therefore, the primary objectives were to evaluate differences in the efficacy in terms of objective tumour responses and overall survival time as well as in the tolerability of the 2 regimens.

### PATIENTS AND METHODS

### Patient selection

Patients between 18 and 70 years of age with advanced metastatic melanoma (according to stage IV; AJCC-classification) (Buzaid et al, 1997) were eligible for the study. Prerequisites for inclusion were a Karnofsky performance status of at least 70%, an expected remaining survival time of at least 3 months, bi-dimensionally measurable disease (bone metastasis and/or efflusions were not considered to be measurable) and the absence of other malignancies. Patients with brain metastasis and/or ocular melanoma were not included. Patients had to be treatment- naive (with the exception of surgical interventions) in stage IV to be included, but previous chemo-, interferon- and/or IL-2 treatment in an adjuvant setting at least 4 weeks prior randomization was not an exclusion criterion. Adequate blood counts (leukocytes >3000/mm$^3$; thrombocytes > 100 000/mm$^3$; haemoglobin level >10 g dl$^{-1}$), renal (creatinine level < 2.0 times the upper limit of normal (ULN) and hepatic functions (bilirubin level < 3 times ULN; AST <3 times ULN) were also required. Patients with active infections, severe underlying diseases (other than metastatic melanoma), manifest thyroid dysfunction, insulin-dependent diabetes mellitus or diseases requiring systemic application of corticosteroids (i.e. autoimmune disorders) were not enrolled. Patients suffering from severe heart diseases including arrhythmia also had to be excluded. Women who were pregnant or breastfeeding were ineligible for the study.

Local ethical review committees of participating centres approved the study. All patients received written information about the purpose of the trial and potential adverse reactions to the study medication. All patients participating in the study gave written informed consent.

### Treatment schedule

Patients were randomized at a central office to receive either DTIC and IFNα2a/b (arm A) or DTIC, IFN- α2a/b plus IL-2 (arm B).

#### *Arm A*

On day one 850 mg/m$^2$ DTIC was administered intravenously (i.v.). Approximately 1 h prior and 1 h following i.v. administration of DTIC 3 MIU/m$^2$ IFN-α2a/b were self-administered subcutaneously (s.c.) by the patient who had previously been instructed in self-injection. From day 2 to 5 patients received 3 MIU/m$^2$ s.c. once daily in the evening. In weeks 2 to 4 IFN-α2a/b was applied 3 times a week in a dose of 3 MIU/m$^2$ s.c. This 4-week regimen was repeated at least twice. To reduce toxicity, serotonin antagonists (i.e. ondansetron, tropisetron, granisetron) were given i.v. prior to chemotherapy. Antipyretics (i.e. paracetamol, metamizol) were applied when necessary at conventional dose levels to overcome cytokine-induced flu-like symptoms.

#### *Arm B*

The same basic medication (DTIC; IFN α2a/b) as in arm A was applied. In addition, IL-2 was administered over different routes (i.v., s.c.) and in varying doses. In the first week IL-2-infusions were started at day 3 with a 3-hour administration of 4.5 MIU/m$^2$ followed by a continuous 24-hour i.v. application of 9.0 MIU/m$^2$ IL-2. From day 4 to 7 patients applied self-injections of 4.5 MIU/m$^2$ IL-2 subcutaneously in the evening. The cumulative dose of IL-2 was 31.5 MIU/m$^2$ per treatment cycle. The side medication to overcome adverse reactions was the same as in arm A, but sometimes had to be administered in higher dosages.

### Pretreatment evaluation

All patients had to provide a complete medical history and be given a thorough physical examination. The following blood tests were performed routinely: blood cell counts, liver, renal and thyroid function tests, electrolytes and serum glucose. These tests were repeated 3 times in week 1 and once a week from weeks 2 to 4. Individual aberrations from these guidelines were tolerated.

In the initial phase of the study an electrocardiogram (ECG) was performed on every patient, later on this procedure was recommended for cardiac risk patients only.

To assess treatment response at least a chest X-ray, an abdominal ultrasound, a CT scan or MRI of the brain and bone scintigraphy were required. Most of the randomized patients also received CT scans of the thorax and abdomen.

### Treatment response re-evaluation

After 8 weeks (2 cycles) of treatment patients were examined by the same physical, biochemical and technical means as in the pretreatment phase. Patients were allowed to continue on protocol as long as the disease was not progressive and blood parameters did not indicate treatment cessation.

Tumour responses (PD, progressive disease; SD, stable disease; PR, partial remission; CR, complete remission) and the severity of adverse events (AEs) were assessed using the criteria of the World Health Organization (WHO).

Patients with non-progressive disease were re-evaluated after every 2 additional cycles of chemoimmunotherapy (following cycle number 4 and 6).

Participating centres were screened by an external monitoring process for the accuracy of submitted patient data, demographics and tumour responses.

### Statistics

The primary objective of this study was to compare the overall survival time of patients in arm A and arm B. Therefore, an intent-to-treat analysis (all randomized patients) and furthermore a ‘per protocol analysis’ by considering only eligible and treated patients was performed. The sample size was calculated on the basis of an

*© 2001 Cancer Research Campaign*

expected difference in survival of 3 months (6 months versus 9 months) with 80% power at a 5% significance level. To calculate overall survival in the 2 treatment arms Kaplan–Meier estimates were generated. Survival curves were compared by means of a log-rank test. Another primary goal of the study was to compare the remission rates achieved by the 2 treatment schedules.

All patients were observed until death or the end of the observation period (November 1999). Data on the exact date and cause of death were recorded. Survival time was calculated from the onset of treatment.

### RESULTS

### Patients

Between October 1994 and April 1998, 290 patients were recruited at 24 centres in Germany and Switzerland (see Appendix). In 7 patients a lost-for-follow-up despite intensive endeavour and in 2 patients a revision of diagnosis (metastatic colorectal cancer instead of metastatic melanoma) became apparent during the external monitoring process in participating centres at the end of the study 281 remaining patients could be evaluated for response and for an intent-to-treat analysis, respectively (Figure 1).

Of these patients 144 were randomized to receive DTIC and IFNα (arm A) and 137 to receive DTIC, IFNα and IL-2 (arm B).

8 patients had to be counted as protocol violators, either because they did not receive treatment in strict accordance with the given protocol or because of violations of the inclusion/exclusion criteria. The reasons for exclusion were previous systemic treatment in stage IV, brain metastasis or non-measurable metastatic sites.

Thus, 273 remaining patients were evaluable for a per-protocol analysis. Of these, 140 patients received DTIC and IFNα (arm A), whereas 133 patients received DTIC, IFNα and IL-2 (arm B).

Patient demographics are presented in Table 1. Analysis of this data revealed no major differences regarding relevant prognostic factors in gender, metastatic sites or the number of metastatic affected organs at baseline between the 2 treatment arms (Pearson chi-square test; $P$ value >0.05).

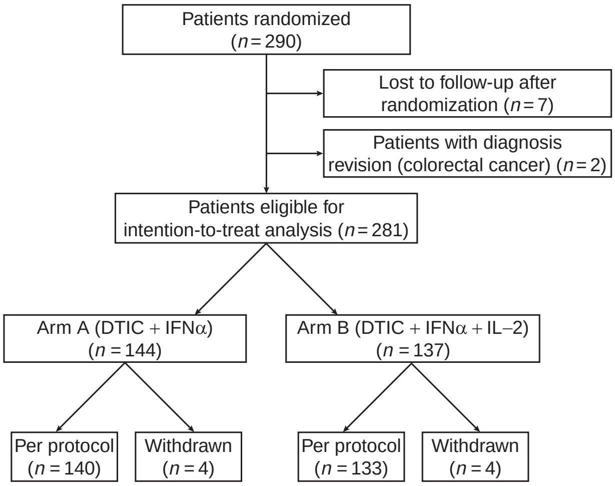

**Figure 1** Disposition of patients

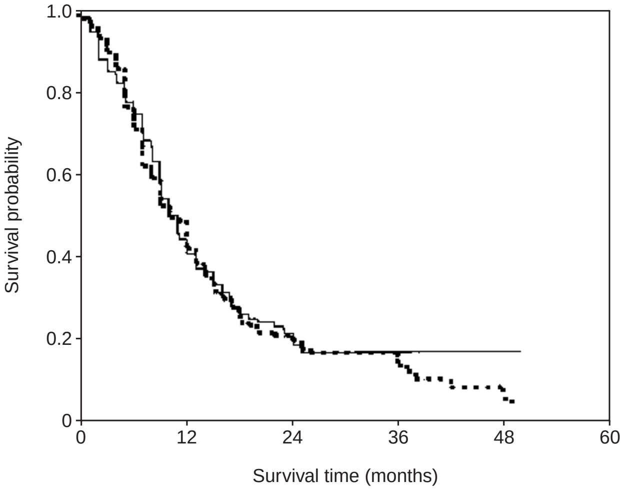

**Figure 2** Kaplan–Meier survival plot of 281 patients randomized based on intent to treat. Median survival time for patients treated with DTIC plus IFNα was 11 months (95% CI: 9.4–12.6 months, continuous line) and for patients treated with DTIC plus IFNα and IL-2 also 11 months (95% confidence interval: 8.9–13.2 months, dotted line, $P$ = 0.52)

### Survival analysis

In the intent-to-treat analysis (ITT) we were able to include survival data on 220 patients (78.3%), who had already died and of 53 patients (18.9%), who were still living at the last monitoring date (censored data). In addition, we included survival data of 8 protocol violators (2.8%).

The overall median survival time was 11 months for arm A patients (DTIC + IFNα) (95% confidence interval: 9.4–12.6) as well as for arm B patients (DTIC + IFNα + IL-2) (95% CI: 8.9–13.2, $P$ = 0.52). The ITT survival curves are shown in Figure 2. There was no statistically significant difference in overall survival between the groups ($P$ = 0.53).

A further statistical evaluation including only the data of patients who were treated according to protocol (‘per-protocol analysis’) also showed no statistical significant difference ($P$ = 0.45) in survival time between the two study arms (arm A: median 11 months, 95% confidence interval: 9.1–12.8 arm B: median 12 months, 95% confidence interval: 9.9–14.1).

### Tumour responses

The ITT analysis of best tumour responses was based on data from 281 patients (144 patients in arm A and 137 patients in arm B). Table 2 shows that tumour responses were almost equally distributed between the 2 treatment groups. 12 melanoma patients (8.3%) receiving arm A treatment (DTIC + IFNα) compared to 10 patients (7.3%) receiving arm B treatment (DTIC + IFNα + IL-2) demonstrated a complete remission (CR) of all measurable metastatic sites. Furthermore, in a total of 26 patients (14 in arm A, 9.7% versus 12 in arm B, 8.8%) partial responses (PR) could be documented. Thus, the overall response rate (CR + PR) for arm A was 18.0% and for arm B 16.1%. The disease remained stable (SD) in 33 patients (22.9%) receiving DTIC and IFN-α and in 32 patients (23.4%) receiving supplementary IL-2 treatment. 58.3% (arm A) and 60.6% (arm B) of the patients respectively suffered from progressive disease (PD) (Table 2). No statistically significant differences in terms of tumour response were detectable between the 2 groups ($P$ = 0.87).

© 2001 Cancer Research Campaign

**Table 1** Patient demographics in 281 evaluable melanoma patients

| | DTIC + IFNα | | DTIC + IFNα + IL-2 | | |
|---|---|---|---|---|---|
| | No. of pts. | % | No. of pts. | % | *P* value |
| Gender | 144 | 51.2 | 137 | 48.8 | 0.66 |
| Male | 95 | 66.0 | 87 | 63.5 | |
| Female | 49 | 34.0 | 50 | 36.5 | |
| Metastatic sites | 141 | 50.9 | 136 | 49.1 | 0.29 |
| Limited disease[a] | 68 | 48.2 | 57 | 41.9 | |
| Extensive disease[b] | 73 | 51.8 | 79 | 58.1 | |
| Number of metastatic organs involved | 141 | 50.9 | 136 | 49.1 | 0.62 |
| 1 | 39 | 27.7 | 45 | 33.1 | |
| 2 | 55 | 39.0 | 49 | 36.0 | |
| 3+ | 47 | 33.3 | 42 | 30.9 | |

[a]Skin and/or lymph node and/or lung involvement. [b]Liver and/or brain and/or bone involvement.

**Table 2** Response rates among 281 assessable patients

| Response | Arm A DTIC + IFNα | | Arm B DTIC + IFNα + IL-2 | |
|---|---|---|---|---|
| | *n* | % | *n* | % |
| Complete remission (CR) | 12 | 8.3 | 10 | 7.3 |
| Partial remission (PR) | 14 | 9.7 | 12 | 8.8 |
| Stable disease (SD) | 33 | 22.9 | 32 | 23.4 |
| Progressive disease (PD) | 84 | 58.3 | 83 | 60.6 |
| Overall response rate (CR + PR) | 26 | 18.1 | 22 | 16.1 |
| Non-progressive patients (CR + PR + SD) | 59 | 41.0 | 54 | 39.4 |

### Toxicity

Potentially, all patients treated per protocol could be assessed in regard to an eventual discontinuation of treatment. However, only 217 patients could be evaluated for whom a detailed analysis of side effects according to the WHO-grading system for adverse events had been performed. For 63 of the patients treated per protocol no detailed WHO-grading for toxicity was reported. This subgroup of patients was evenly distributed between the 2 treatment arms.

In general, both treatment modalities were well tolerated and most of the adverse events were only mild or moderate (WHO grades I/II). The most common side effects were, as expected, flu-like symptoms such as fever, chills or fatigue. These reactions were more frequently observed in the treatment group receiving IL-2 (Table 3).

Patients in arm B suffered more often from nausea and vomiting, somnolence, diarrhoea, dyspnoea, pruritic skin rash and alopecia. Grade 3 and 4 toxicities were observed only occasionally (in 1 to 2% of the patients) and were reversible after discontinuation of the study drug. No life-threatening adverse events were reported.

Haematological toxicity in the form of mild myelosuppression was frequently observed, but grade III reactions (up to 10%) and grade IV toxicity (up to 3%) rarely occurred (Table 3). Haematological toxicity was reversible in all cases.

Differences in the frequency and severity of haematological adverse events between the 2 treatment groups were not

**Table 3** Adverse reactions observed among 217 patients evaluated for toxicity

| Adverse event | Arm A: DTIC + IFNα WHO-grade (%) | | | Arm B: DTIC + IFNα + IL-2 WHO-grade (%) | | |
|---|---|---|---|---|---|---|
| | I/II | III | IV | I/II | III | IV |
| Anaemia | 26 | 1 | 0 | 36 | 1 | 3 |
| Leucopenia | 38 | 10 | 0 | 41 | 3 | 3 |
| Neutropenia | 32 | 6 | 0 | 28 | 1 | 3 |
| Thrombocytopenia | 21 | 4 | 2 | 19 | 3 | 3 |
| Elevated liver enzymes | 26 | 3 | 1 | 31 | 4 | 1 |
| Elevated creatinine | 3 | 0 | 0 | 6 | 2 | 0 |
| Fever/chills | 52 | 2 | 1 | 75 | 9 | 2 |
| Nausea/vomiting | 44 | 8 | 0 | 55 | 14 | 2 |
| Somnolence | 4 | 1 | 0 | 20 | 0 | 0 |
| Obstipation | 7 | 0 | 0 | 9 | 0 | 0 |
| Diarrhoea | 12 | 1 | 0 | 20 | 3 | 0 |
| Dyspnoea | 4 | 0 | 1 | 9 | 2 | 0 |
| Skin rash | 10 | 0 | 0 | 32 | 0 | 0 |
| Alopecia | 13 | 1 | 0 | 22 | 1 | 0 |

© 2001 Cancer Research Campaign 

detectable. However, treatment had to be discontinued because of side effects more often for patients receiving supplementary IL-2 (arm B) than for those receiving only DTIC and IFNα ($P = 0.043$) and this difference remained constant for all of the documented side effects. In arm B treatment of 19 (13.9%) patients had to be discontinued due to side effects of biochemotherapy whereas these side effects were severe enough to necessitate discontinuation in only 8 (5.6%) of the arm A patients. In addition, a small number of patients (arm A, $n = 1$, 0.7% versus arm B, $n = 3$; 2.2%) refused further treatment for personal reasons, not because they suffered from significant non-haematological or haematological side effects.

In the arm A patient population 53.9% received 1 to 3 treatment cycles, 27.0% 4 to 6 cycles and 19.1% more than 6 cycles of the given treatment. In contrast, the number of applied treatment cycles in the patient population treated with IL-2 (arm B) was significantly lower ($P = 0.028$). 63% of patients received 1 to 3, 28.9% 4 to 6 and 8.1% more than 6 treatment cycles.

One patient committed suicide in the face of increasing disability due to progressive melanoma causing paraplegia. This was not considered to be related to the study drugs.

### DISCUSSION

In this prospective-randomized multicentre phase III-trial we compared survival data, tumour responses and the toxicity profiles in patients receiving 2 treatment schedules. One treatment group (arm A) received DTIC and IFNα, a second group (arm B) received the same plus IL-2. To our knowledge, this study represents the first randomized trial on the efficacy of a biochemotherapy regimen with or without the addition of IL-2.

Up to the present, IL-2 has either been applied as a monotherapy in several single-institutional studies with remission rates of about 15 to 20% (Atkins et al, 1999) or as an adjunct to chemotherapy (i.e. DTIC) (Dummer et al, 1995) or to IFNα (Keilholz et al, 1998). In the last years, several phase II-trials have focused on biochemotherapy consisting of a combination of several cytotoxic substances with IFNα and IL-2 (Legha et al, 1998; Proebstle et al, 1998; Richards et al, 1999; Sertoli et al, 1999). Legha and co-workers reported on encouraging tumour responses (21% CR, 43% PR) achieved with biochemotherapy in advanced metastatic melanoma patients (Legha et al, 1998). They used cisplatin, vinblastine, DTIC plus IL-2 (i.v. administration) and IFNα (s.c. administration). Richards and colleagues reported on quite similar results (55% response rate) with a combination of cisplatin, DTIC and carmustine with i.v. IL-2 and s.c. IFNα in 83 metastatic melanoma patients. Both authors recommended performing a randomized trial to establish the value of biochemotherapy for melanoma treatment in the future (Legha et al, 1998; Richards et al, 1999). Recently, Rosenberg and co-workers (Rosenberg et al, 1999) published data on a prospective-randomized trial using chemotherapy with cisplatin, dacarbazine and tamoxifen alone or in combination with IL-2 and IFNα2b. 102 patients were enrolled in this National Cancer Institute study. However, the investigators discovered that the addition of immunotherapy to chemotherapy increased toxicity, but did not enhance survival. Despite the better response rate for patients who had received biochemotherapy (44% versus 27%), the survival rate was better for patients receiving chemotherapy only (15.8 months versus 10.7 months). Rosenberg and colleagues concluded that biochemotherapy cannot be recommended until well-designed, prospective protocols have demonstrated the benefits of this treatment strategy (Rosenberg et al, 1999). Interestingly, no randomized study on biochemotherapy published up to now has focused on the addition of IL-2 as the sole variable despite recommendations in the literature (Keilholz, 1995).

Our study could demonstrate neither an increase in response rates (CR/PR) nor a prolongation of the survival time when IL-2 was added to DTIC and IFNα. The response rate for DTIC and IFNα without IL-2 (18.0%) corresponds to that reported in previously published phase III trials (Falkson et al, 1998). Surprisingly, we failed to demonstrate an expected higher rate of responses with the addition of IL-2 (16.2%). One explanation for these findings might be that IL-2 is inactive in this setting. The cumulative dose of IL-2 in our study (31.5 MIU/$m^2$) is low compared to high-dose schedules used in other trials (Keilholz et al, 1998; Legha et al, 1998; Richards et al, 1999). Furthermore, we used an IL-2 administration scheme adapted from the ‘decrescendo schedule’ introduced by Keilholz and co-workers (Keilholz et al, 1997). In the US-trials continuous infusions with constant doses of IL-2 were preferred (Legha et al, 1998; Richards et al, 1999; Rosenberg et al, 1999). In our study, IL-2 was administered intravenously on days 3 and 4 only, on the subsequent days the subcutaneous route of administration was chosen to allow patients self-administration of IL-2 at home. Although efficacy of subcutaneous IL-2 was demonstrated in several combination studies with chemotherapy and/or IFNα (Atzpodien et al, 1990; Hoffmann et al, 1998; Kashani-Sabet et al, 1999), data on the activity of IL-2 applied subcutaneously as a monotherapy are limited (Atzpodien et al, 1990; Schomburg et al, 1992).

Dreno and co-workers (Dreno et al, 1995) reported on a quite similar chemoimmunotherapy scheme comparable to our study. They observed 2 complete and 3 partial responders (response rate: 26.2%) among 19 metastatic melanoma patients. All patients received DTIC, IFNα2a and subcutaneous IL-2 in an outpatient setting (Dreno et al, 1995).

In regard to the toxicity profile significant differences between the treatment groups with or without IL-2 application were documented in our study. However, we have to consider that most of the adverse reactions were grade I and II toxicities while grade III and IV side effects occurred infrequently. Thus, it was surprising that in the population of patients receiving IL-2 treatment discontinuation was documented in 13.9% of affected patients in contrast to 5.6% of the patients who received no IL-2. We have to carefully consider the possibility that the higher rate of treatment discontinuation and the lower number of treatment cycles carried out in patients receiving IL-2 may have been due to the lack of experience in applying and dealing with the side effects of IL-2 in some of the centres participating in this study. To avoid these problems, the Dermatologic Cooperative Oncology Group (DeCOG) offered training programmes (‘management of adverse reactions’) for participating centres during the whole study period. In general, the toxicity profile for DTIC and IFNα with or without IL-2 turned out as was expected. The study medication was well tolerated by most patients in both arms. The majority of patients received more than 90% of the scheduled dose of both cytokines. Life-threatening adverse reactions related to therapy were not observed.

In conclusion, this study was not able to demonstrate that the addition of IL-2 to DTIC and IFNα resulted in an increased response rate or a longer overall survival time. Interestingly, the overall survival time of our entire collective with a median of 11 months was quite good in comparison with other results reported

© 2001 Cancer Research Campaign in the literature. The toxicity of both regimens was in general low to moderate. However, significantly more side effects and treatment discontinuations were observed in the patient population receiving IL-2. Consequently, it still seems questionable that IL-2 regimens with moderate dosages improve biochemotherapy schedules for metastatic melanoma. The benefits of continuous high-dose infusions of IL-2 in this context still have to be determined. The European Organisation for Research and Treatment of Cancer (EORTC) has designed a study in order to test the high-dose decrescendo schedule and results will be available soon.

### ACKNOWLEDGEMENTS

The following companies provided grants for documentation, study office staff, and independent monitoring of participating centres: Chiron Therapeutics (Ratingen/Germany), Essex Pharma (Munich/Germany) and Hoffmann-La Roche (Grenzach-Wyhlen/Germany).

### REFERENCES

Atkins MB, Lotze MT, Dutcher JP, Fisher RI, Weiss G, Margolin K, Abrams J, Sznol M, Parkinson D, Hawkins M, Paradise C, Kunkel L and Rosenberg SA (1999) High-dose recombinant interleukin 2 therapy for patients with metastatic melanoma: analysis of 270 patients treated between 1985 and 1993. *J Clin Oncol* **17**: 2105–2111

Atzpodien J, Korfer A, Evers P, Franks CR, Knuver-Hopf J, Lopez-Hanninen E, fischer M, Mohr H, Dallmann I and Hadam M (1990a) Low-dose subcutaneous recombinant interleukin-2 in advanced human malignancy: a phase II outpatient study. *Mol Biother* **2**: 18–26

Atzpodien J, Korfer A, Franks CR, Poliwoda H and Kirchner H (1990b) Home therapy with recombinant interleukin-2 and interferon-alpha 2b in advanced human malignancies. *Lancet* **335**: 1509–1512

Buzaid AC, Ross MI, Balch CM, Soong S, McCarthy WH, Tinoco L, Mansfield P, Lee JE, Bedikian A, Eton O, Plager C, Papadpoulos N, Legha SS and Benjamin RS (1997) Critical analysis of the current American Joint Committee on Cancer staging system for cutaneous melanoma and proposal of a new staging system. *J Clin Oncol* **15**: 1039–1051

Chapman PB, Einhorn LH, Meyers ML, Saxman S, Destro A, Panageas K, Begg C, Agarwala S, Schluchter L, Ernsthoff M, Houghton A and Kirkwood J (1999) Phase III multicenter randomized trial of the Dartmouth regimen versus dacarbazine in patients with metastatic melanoma. *J Clin Oncol* **17**: 2745–2751

Dréno B, Cupissol D, Joly P (1995) Ambulatory treatment of metastatic melanoma associating subcutaneous dacarbazine, interferon α and interleukin-2. JEADV **4**: 248–253

Dummer R, Gore ME, Hancock BW, Guillou PJ, Grobben HC, Becker JC, Oskam R, Dieleman JP and Burg G (1995) A mulicenter phase II clinical trial using dacarbazine and continuous infusion interleukin-2 for metastatic melanoma. Clinical data and immunomonitoring. *Cancer* **75**: 1038–1044

Falkson CI, Falkson G and Falkson HC (1991) Improved results with the addition of interferon alfa-2b to dacarbazine in the treatment of patients with metastatic malignant melanoma. *J Clin Oncol* **9**: 1403–1408

Falkson CI, Ibrahim J, Kirkwood JM, Coates AS, Atkins MB and Blum RH (1998) Phase III trial of dacarbazine versus dacarbazine with tamoxifen versus dacarbazine with interfron alpha-2b and tamoxifen in patients with metastatic malignant melanoma: an Eastern Cooperative Oncology Group study. *J Clin Oncol* **16**: 1743–1751

Grob JJ, Dreno B, de la Salmoniere P, Delauny M, Cupissol D, Guillot B, Souteyrand P, Sassolas B, Cesarini JP, Lionnet S, Lok C, Chastang C and Bonerandi JJ (1998) Randomised trial of interferon alpha-2a as adjuvant therapy in resected primary melanoma thicker than 1.5 mm without clinically detectable node metastases. French Cooperative Group on Melanoma. *Lancet* **351**: 1905–1910

Hill GJ 2d, Krementz ET and Hill HZ (1984) Dimethyl triazeno imidazole carboxamide and combination therapy for melanoma. IV. Late results after complete response to chemotherapy (Central Oncology Group protocols 7130, 7131, and 7131A). *Cancer* **53**: 1299–1305

Hoffmann R, Müller I, Neuber K, Lassmann S, Buer J, Probst M, Oevermann K, Franzke A, Kirchner H, Ganser A and Atzpodien J (1998) Risk and outcome in metastatic malignant melanoma patients receiving DTIC, cisplatin, BCNU and tamoxifen followed by immunotherapy with interleukin 2 and interferon alpha2a. *Br J Cancer* **78**: 1076–1080

Kashani-Sabet M, Sagebiel RW, Collins HE, Glassberg AB, Allen RE, Leong SP and Small EJ (1999) Outpatient combination chemoimmunotherapy for patients with metastatic melanoma. Results of a phase I/II trial. *Cancer* **86**: 2160–2165

Keilholz U (1995) Chemoimmunotherapy of melanoma. Is it time for phase III trials? *Cancer* **75**: 905–907

Keilholz U, Goey SH, Punt CJ, Proebtsle TM, Salzmann R, Scheibenbogen C, Schadendorf D, Lienard D, Enk A, Dummer R, Hantich B, Geueke AM and Eggermont AM (1997) Interferon alfa-2a and interleukin-2 with or without cisplatin in metastatic melanoma: a randomized trial of European Organization for Research and Treatment of Cancer Melanoma Cooperative Group. *J Clin Oncol* **15**: 2579–2588

Keilholz U, Conradt C, Legha SS, Khayat D, Scheibenbogen C, Thatcher N, Goey SH, Gore M, Dorval T, Hancock B, Punt JC, Dummer R, Avril MF, Brocker EB, Benhammouda A, Eggermont AM and Pritsch M (1998) Results of interleukin-2-based treatment in advanced melanoma: a case record-based analysis of 631 patients. *J Clin Oncol* **16**: 2961–2969

Kirkwood JM, Strawdermann MH, Ernstoff MS, Smith TJ, Borden EC and Blum RH (1996) Interferon alfa-2b adjuvant therapy of high-risk resected cutaneous melanoma: the Eastern Cooperative Oncology Group Trial EST 1684. *J Clin Oncol* **14**: 7–17

Legha SS, Ring S, Eton O, Bedikian A, Buzaid A, Plager C and Papadopoulos N (1998) Development of a biochemotherapy regimen with concurrent administration of cisplatin, vinblastine, dacarbazine, interferon alfa, and interleukin-2 for patients with metastatic melanoma. *J Clin Oncol* **16**: 1752–1759

Middleton MR, Grob JJ, Aaronson N, Fierlbeck G, Tilgen W, Seiter S, Gore M, Aamdal S, Cebon J, Coates A, Dreno B, Henz M, Schadendorf D, Kapp A, Weiss J, Fraass U, Statkevich P, Muller M and Thatcher N (2000) Randomized phase III study of temozolomide versus dacarbazine in the treatment of patients with advanced metastatic malignant melanoma. *J Clin Oncol* **18**: 158–166

Pehamberger H, Soyer HP, Steiner A, Kofler R, Binder M, Mischer P, Pachinger W, Aubock J, Fritsch P, Kerl H and Wolff K (1998) Adjuvant interferon alfa-2a treatment in resected primary stage II cutaneous melanoma. Austrian Malignant Melanoma Cooperative Group. *J Clin Oncol* **16**: 1425–1429

Proebstle TM, Fuchs T, Scheibenbogen C, Sterry W and Keilholz U (1998) Long-term outcome of treatment with dacarbazine, cisplatin, interferon-alpha and intravenous dose interleukin-2 in poor risk melanoma patients. *Melanoma Res* **8**: 557–563

Punt CJ (1998) The use of interferon-alpha in the treatment of cutaneous melanoma: a review. *Melanoma Res* **8**: 95–104

Richards JM, Gale D, Mehta N and Lestingi T (1999) Combination of chemotherapy with interleukin-2 and interferon alfa for the treatment of metastatic melanoma. *J Clin Oncol* **17**: 651–657

Rosenberg SA, Yang JC, Schwartzentruber DJ, Hwu P, Marincola F, Topalian S, Seipp C, einhorn J, White D, Steinberg (1999) Prospective randomized trial of the treatment of patients with metastatic melanoma using chemotherapy with cisplatin, dacarbazine, and tamoxifen alone or in combination with interleukin-2 and interferon alfa-2b. *J Clin Oncol* **17**: 968–975

Schomburg A, Menzel T, Korfer A, Heer G, Dallmann I, Kirchner H, Poliwoda H, Atzpodien J (1992) In vivo and ex vivo antitumor activity in patients receiving low-dose subcutaneous recombinant interleukin-2. *Nat Immun* **11**: 133–143

Sertoli MR, Queirolo P, Bajetta E, DelVecchio M, Comella G, Barduagni L, Bernengo MG, Vecchio S, Criscuolo D, Bufalino R, Morabito A and Cascinelli N (1999) Multi-institutional phase II randomized trial of integrated therapy with cisplatin, dacarbazine, vindesine, subcutaneous interleukin-2, interferon alpha2a and tamoxifen in metastatic melanoma. BREMIM (Biological Response Modifiers in Melanoma). *Melanoma Res* **9**: 503–504

*© 2001 Cancer Research Campaign* 

### APPENDIX: PARTICIPATING CENTERS AND INVESTIGATORS

| Centre | Investigators |
|---|---|
| Aarau[1] | M Wernli |
| Bern[1] | M Fey, A Barth |
| Buxtehude[2] | P Mohr |
| Dresden[2] | G Sebastian, U Köhler |
| Duisburg[2] | J Kunze, T Hendricks |
| Erfurt[2] | R Linse, Y Kellner |
| Flensburg[2] | M Dürk |
| Frankfurt[2] | R Kaufmann, D Rinne, K Spieth |
| Freiburg[2] | K Mross, J Arends |
| Hagen[2] | R Souchon |
| Heidelberg[2] | S Seiter, K Uhl, H Näher |
| Homburg[2] | S Ugurel, S Seiter, W Tilgen |
| Kiel[2] | M Möller, A Hauschild, E Christophers |
| Köln[2] | T Krieg, S Theile-Ochel |
| Lübeck[2] | W Achtelik, HH Wolff |
| Magdeburg[2] | J Ulrich, H Gollnick |
| Mannheim[2] | R Rzany, B Roth, A Stein |
| München[2] | M Volkenandt |
| Münster[2] | D Nashan, M Schiller |
| Regensburg[2] | W Stolz, I Langenbach |
| Tübingen[2] | C Garbe, P Radny |
| Würzburg[2] | EB Bröcker, I Kerstings |
| Zürich[1] | R Dummer, L Heinzerling |

[1]Switzerland, [2]Germany.

 *© 2001 Cancer Research Campaign*
